# Splenic ischemic preconditioning attenuates oxidative stress induced by hepatic ischemia-reperfusion in rats^1^

**DOI:** 10.1590/s0102-865020190070000007

**Published:** 2019-09-16

**Authors:** Caio César Chaves Costa, Nathalia Gabay Pereira, Anna Luiza Melo Machado, Mariana Albuquerque Dórea, Rafaella Macêdo Monteiro da Cruz, Renata Cunha Silva, Robson José de Souza Domingues, Edson Yuzur Yasojima

**Affiliations:** I Graduate student, Faculty of Medicine, UEPA, Belem-PA, Brazil. Technical procedures, analysis and interpretation of data, manuscript preparation; II Fellow, Postgraduate Program in Surgery and Experimental Research, UEPA, Belem-PA, Brazil. Technical procedures, analysis and interpretation of data, manuscript preparation; III PhD, Full Professor, Department of Morphology and Physiological Sciences, UEPA, Belem-PA, Brazil. Scientific and intellectual content of the study, critical revision, final approval; IV PhD, Full Professor, Postgraduate Program in Surgery and Experimental Research, Universidade do Estado do Pará (UEPA), Belem-PA, Brazil. Conception, design, scientific and intellectual content of the study; critical revision; final approval

**Keywords:** Liver Failure, Reperfusion Injury, Oxidative Stress, Ischemic Preconditioning, Rats

## Abstract

**Purpose:**

To evaluate the effects of splenic ischemic preconditioning (sIPC) on oxidative stress induced by hepatic ischemia-reperfusion in rats.

**Methods:**

Fifteen male Wistar rats were equally divided into 3 groups: SHAM, IRI and sIPC. Animals from IRI group were subjected to 45 minutes of partial liver ischemia (70%). In the sIPC group, splenic artery was clamped in 2 cycles of 5 min of ischemia and 5 min of reperfusion (20 min total) prior to hepatic ischemia. SHAM group underwent the same surgical procedures as in the remaining groups, but no liver ischemia or sIPC were induced. After 1h, hepatic and splenic tissue samples were harvested for TBARS, CAT, GPx and GSH-Rd measurement.

**Results:**

sIPC treatment significantly decreased both hepatic and splenic levels of TBARS when compared to IRI group (p<0.01). Furthermore, the hepatic and splenic activities of CAT, GPx and GSH- Rd were significantly higher in sIPC group than in IRI group.

**Conclusion:**

sIPC was able to attenuate hepatic and splenic IRI-induced oxidative stress.

## Introduction

Ischemia-reperfusion injury (IRI) refers to an exacerbation of cellular damage following restoration of blood flow and oxygen delivery to hypoxic tissues^1^. It is a common cause of liver dysfunction after major resection/transplantation^2^ and contributes to a high morbidity and mortality. Pathogenic mechanisms implicated in early hepatic IRI include Kupffer cell activation, release of proinflammatory cytokines and oxidative stress due to the overproduction of reactive oxygen species (ROS)^2^ ^,^ ^3^.

Ischemic preconditioning (IPC), defined as multiple brief ischemic episodes before a sustained ischemic insult^4^, has been shown to attenuate experimental liver IRI by decreasing oxidative damage, inflammation, apoptosis and microcirculatory dysfunction^5^. When applied to tissues that are not directly exposed to ischemia, IPC can also provide cellular protection against a distant organ IRI, which is known as remote ischemic preconditioning (RIPC) procedure^4^. Different anatomical sites have been successfully used to perform RIPC among rodent models of hepatic IRI, such as hindlimb^6^ and superior mesenteric artery^7^, but other locations may ensure favorable outcomes as well.

Evidences suggest that spleen plays a pivotal role in the pathophysiology of liver IRI and that interventions modulating its function may be important therapeutic strategies. For example, splenic congestion during hepatic IRI can promote splenic interleukins excretion with subsequent leukocytes infiltration and parenchyma damage within liver^8^. Thus, splenectomy^9^ or splenic artery ligation^10^ performed prior to hepatic IRI have been able to suppress this collateral reaction and reduce both biochemical and histopathological injury. Interestingly, a recent study demonstrated that intermittent occlusion of the splenic pedicle, described as splenic ischemic preconditioning (sIPC), can remotely reduce a remote renal IRI^11^; however, there is no current data reporting its effects on the liver. Considering that RIPC protocols have a protective effect against hepatic IRI, transient splenic ischemia through sIPC could be also a hepatoprotective intervention.

Therefore, in this study, we aimed to investigate the effects of sIPC on oxidative stress induced by liver IRI in rats.

## Methods

All experiments were approved by the Ethics Committee for the Use of Animals, Universidade do Estado do Pará (protocol number: 20/17) and followed the rules of the Brazilian National Law for Animal Care (Law 11.794/08).

A total of 15 male Wistar rats (12-15 weeks), weighing 260-310 g, were obtained from the vivarium of the Laboratory of Experimental Surgery (Pará State University, Brazil). Rats were kept under constant environmental conditions with controlled temperature (22°C-24°C), 12h day-night cycles and food/water *ad libitum.*

The animals were randomly allocated into three experimental groups: the IRI group underwent 45 minutes of liver ischemia followed by 60 minutes of reperfusion (n=5); the sIPC group was submitted to intermittent clamping of splenic artery (2 cycles of 5 min of ischemia and 5 min of reperfusion) prior to liver ischemia (n=5); and the sham-operated (SHAM) group underwent the same surgical procedures as in the remaining groups, but no liver ischemia or sIPC was induced (n=5).

### Surgical procedures

For the surgical operation, all animals were anesthetized with intraperitoneal injections of ketamine (70 mg/kg) and xylazine (10 mg/kg); then, they were placed on a heating pad to maintain body temperature between 36.5-37.5°C. Through a midline laparotomy, partial hepatic ischemia (70% of liver mass) was induced by clamping the portal vein, hepatic artery and bile duct supplying the median and left lobes with an atraumatic microvascular clamp. In this method, the blood supply to right and caudate lobes remained uninterrupted, attenuating intestinal congestion through portal flow bypass^12^. Reperfusion was initiated by removing the microvascular clamp. During all procedure, the bowel was protected by a gauze with heated saline (NaCl 0.9%) in order to avoid fluid loss through evaporation. Liver IRI was produced for 45 minutes of ischemia followed by 60 minutes of reperfusion^13^. After that period, left and median hepatic lobes, as well as the spleen, were harvested for biochemical analysis. Lastly, the animals were euthanized by lethal anesthetic doses.

In the sIPC group, splenic artery was dissected from its origin situated posterior to stomach. The gastrosplenic ligament, which contains short gastric vessels, remained intact and the splenocolic ligament was cut to facilitate spleen manipulation. sIPC was performed through intermittent occlusion of the splenic artery with an atraumatic microvascular clamp^11^ and consisted in 2 cycles of 5 minutes of ischemia and 5 minutes of reperfusion prior to the induction of liver ischemia.

### Assay of TBARS concentration

Concentration of thiobarbituric acid reactive substances (TBARS), which represents the extent of lipid peroxidation by free-radicals, was measured using Winterbourn method^14^ modified to spectrophotometry analysis. Briefly, a mixture containing 0.2 ml of homogenized tissue sample (liver or spleen), 0.02 ml of butylated hydroxy toluene (BHT), 0.2 ml of 25% hydrochloric acid (HCl) and 0.2 ml of thiobarbituric acid (TBA) solution was vortexed and then heated at 100°C for 15 minutes. After cooling, 0.618 ml of butanol was added and centrifuged at 4.000 rpm for 2 minutes. Subsequently, the supernatant was taken out and its absorbance was measured at 532 nm. TBARS were quantified using an extinction coefficient of 1.56 × 10^5^ mol^−1^.cm^−1^ and expressed as nmol of TBARS per milliliters (nmol/ml).

### Assay of CAT, GPx and GSH-Rd activities

Catalase (CAT) activity was measured according to the Aebi method^15^. An aliquot of tissue homogenate (0.2 ml) was added to a cuvette containing sodium phosphate buffer (pH 7) and 10 mM hydrogen peroxide (H_2_O_2_). Then, the enzymatic decomposition of hydrogen peroxide was monitored as the decrease in absorbance at 240 nm for 20 seconds and the results were expressed as units per milliliters (U/ml).

Glutathione peroxidase (GPx) activity was determined using the method described by Flohé and Gunzler^16^ which is based on the reduction of tert-butyl hydroperoxide by the oxidation of glutathione (GSH) and formation of oxidized glutathione (GSSG) catalyzed by GPx. Subsequently, the decrease in absorbance of nicotinamide adenine dinucleotide phosphate (NADPH) was analyzed at 340 nm, since NADPH is used in the restoration of GSH. The results were expressed as U/ml.

Glutathione reductase (GSH-Rd) activity was determined spectrophotometrically by measuring the rate of NADPH oxidation, due to GSH formation from GSSG, at 340 nm according to Carlberg and Mannervik protocol^17^. GSH-Rd concentration was represented as U/ml.

### Statistical analysis

Results were expressed as means ± standard deviation and analyzed using SPSS software version 25.0 for Windows (IBM Corp., Armonk, NY, USA). Shapiro-Wilk test was used to verify the normal distribution of the data. One-way analyses of variance with Tukey *post hoc* or Kruskal-Wallis test were performed to assess differences between groups. Statistical significance was assumed at p<0.05.

## Results

**Figure 1:**
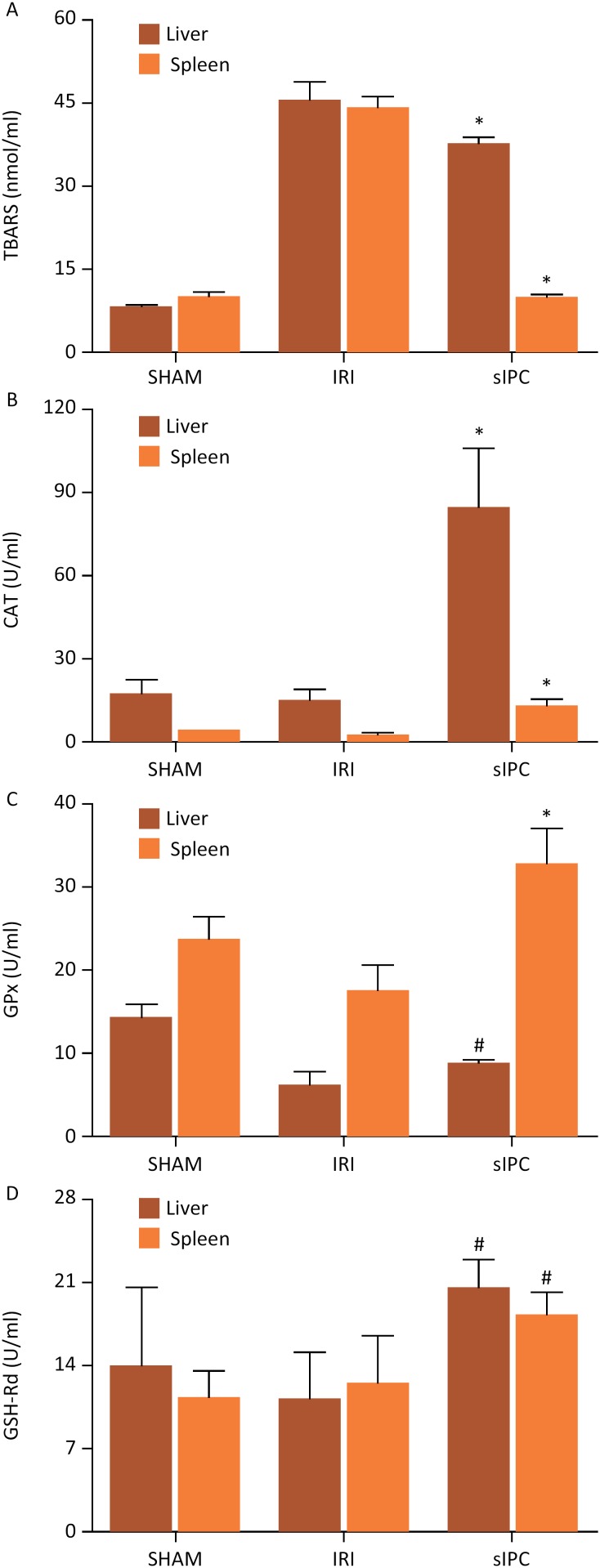
Hepatic and splenic levels of TBARS (**A**), CAT (**B**), GPX (**C**) and GSH-Rd (**D**) according to groups (n = 5 for each). Data are presented as mean ± standard deviation. *p<0.01, sIPC vs. IRI; #p<0.05, sIPC vs. IRI.

IRI group had significantly higher hepatic (45.29 ± 3.18) and splenic (43.74 ± 2.14) levels of TBARS when compared to SHAM group (liver: 7.95 ± 0.57, *P* < 0.01; spleen: 9.99 ± 0.91, p<0.01). However, sIPC markedly decreased TBARS in liver (37.75 ± 0.94) and spleen (9.94 ± 0.63) as compared to IRI group (p<0.01) (Fig. 1A). CAT concentration was lower in IRI group (liver: 15.10 ± 3.44; spleen: 2.36 ± 0.23) than the SHAM group (liver: 17.53 ± 4.47; spleen: 4.20 ± 0.28), but without statistical difference. Rats from sIPC group showed remarkably higher CAT levels (liver: 84.65 ± 20.77; spleen: 13.05 ± 2.03) than those from IRI group (p<0.01) (Fig. 1B). GPx levels were significantly lower in IRI group (liver: 6.06 ± 1.66; spleen: 17.51 ± 2.86) than the SHAM group (liver: 14.35 ± 1.41, p<0.01; spleen: 23.57 ± 2.82, p<0.05). Notably, compared with the IRI group, sIPC increased GPx levels (liver: 8.81 ± 0.33, p<0.05; spleen: 32.65 ± 4.17, p<0.01) (Fig. 1C). Lastly, IRI mildly reduced GSH-Rd levels (liver: 11.20 ± 3.84; spleen: 12.44 ± 3.95) when compared to SHAM group (liver: 13.85 ± 6.65; spleen: 11.26 ± 2.21), although there was no statistical distinction. In contrast, GSH-Rd levels were significantly higher in sIPC group (liver: 20.51 ± 2.33; spleen: 18.29 ± 1.79) than in the IRI group (p<0.05) (Fig. 1D).

## Discussion

To our knowledge, this is the first study to investigate the beneficial effects of sIPC in an experimental model of liver IRI. Our experiment was inspired by the previous report by Shen *et al.* ^11^, which successfully demonstrated an anti-inflammatory activity of sIPC against renal IRI in rats. In their study, sIPC was achieved through intermittent clamping of the splenic pedicle and 3 cycles of 5 minutes of ischemia and 5 minutes of reperfusion were used. In our sIPC protocol, for the purpose of minimizing surgical trauma on the splenic vasculature, we only performed 2 cycles of 5 minutes of ischemia and 5 minutes of reperfusion.

In summary, our results showed that sIPC treatment suppressed oxidative stress induced by IRI, which was demonstrated by decreased TBARS level and enhanced activity of CAT, GPx and GSH-Rd. Moreover, we established that spleen is one of the organs affected by liver IRI, since rats submitted to IRI presented higher splenic levels of TBARS in association with lower concentration of CAT, GPx and GSH-Rd when compared to SHAM.

It is known that overproduction of the ROS is one of the main pathological process implicated in hepatic IRI. During the ischemic period, the reduction of oxygen supply culminates in adenosine triphosphate (ATP) depletion and failure of active transmembrane ion-transport, which sequentially leads to endothelial swelling and microcirculatory dysfunction^2^ ^,^ ^3^. Posteriorly, there is activation of Kupffer cells with subsequent release of inflammatory cytokines and ROS, such as hydroxyl (OH^−^), superoxide anion (O_2_^−^) and hydrogen peroxide (H_2_O_2_)^3^. Concomitantly, hypoxanthine accumulates in hepatic tissue due to progressive catabolism of ATP^18^.

Once reperfusion occurs, the restoration of oxygen delivery paradoxically exacerbates the initial insult through increased activation of enzymes involved in free-radicals production, such as xanthine-oxidase (XO), which in the presence of oxygen catalyzes the conversion of hypoxanthine to xanthine and simultaneously generates superoxide^1^ ^,^ ^18^. The immediate consequence is characterized as an accumulation of ROS, resulting in extensive hepatocyte damage through DNA oxidation and lipid peroxidation^3^.

Endogenous antioxidant enzymes represent the major tissue defense against oxidative stress induced by IRI, since they are capable of removing oxygen free-radicals before they harm cellular structures^19^. In our study, we assessed the antioxidant activity of hepatic parenchyma by measuring the concentration of the enzymes CAT, GPx and GSH-Rd. CAT catalyzes the reduction of H_2_O_2_ to H_2_O and O_2_, preventing its participation in the formation of OH^−^, a highly deleterious free-radical; GPx reduces H_2_O_2_ and organic peroxides while oxidizing GSH to GSSG^19^; and GSH-Rd maintains a high GSH/GSSG ratio by reducing GSSG and restoring GSH, which is a defensive mechanism against oxidative stress^20^.

It has been described that liver IRI is linked to depletion of these enzymes^21^. On the other hand, their increase is associated with favorable outcomes of hepatic IPC^22^, which was probably one of the cytoprotective effects induced by sIPC in our experiment. In 2002, Sindram *et al.* ^23^ suggested that oxygen-radicals play a pivotal role in triggering hepatoprotection from IPC. In their study, pretreatment with N-acetylcysteine (NAC), an effective free-radical scavenger, reversed the beneficial effects of IPC, indicating an oxidative stress dependent mechanism. Later evidences have shown that ROS exposure can also upregulate liver antioxidant systems by activating transcription factors within hepatocytes^24^. Therefore, we hypothesize that our sIPC protocol provoked a short sublethal burst of oxygen free-radicals into circulation, which remotely enhanced antioxidant activity in hepatic tissue.

In general, the protective effects mediated by RIPC are not fully understood yet and other mechanisms may also have occurred in our study. For example, previous evidences reported that RIPC produced by transient limb^6^ or intestinal^7^ ischemia can attenuate liver IRI by inducing hepatic expression of Heme-Oxygenase-1 (HO-1), an enzyme responsible to degrade heme. HO-1 is also able to scavenge free-radicals and is commonly upregulated during stress conditions, such as hypoxia or oxidative stress^25^.

We suggest that ROS released by sIPC could be taken to the liver and stimulated the local production of HO-1, which mediated a defensive effect against IRI. In addition, since HO-1 is normally detected in rat's spleen^10^, the transient hypoxia induced by our sIPC could upregulate its local expression, allowing its eventual drainage to the hepatic tissue. Furthermore, in the study of Shen *et al*.^11^, sIPC attenuated renal IRI through inhibition of NF-kB pathway and upregulation of interleukin-10 (IL-10) expression, indicating an antiinflammatory effect. NF-kB is a nuclear transcription factor that regulates the expression of several proinflammatory cytokines and its suppression are associated with beneficial effects during liver IRI^2^. Interestingly, RIPC performed through intermittent occlusion of superior mesenteric artery can also reduce the activity of NF-kB in the liver^7^, which implies a probable common hepatoprotective effect of RIPC procedures, including our sIPC protocol.

Current data indicate that RIPC induces tissue protection through a systemic neurohormonal connection. This mechanism is based on the release of humoral mediators or stimulation of afferent innervation that are capable of activating cellular survival pathways in a remote tissue^4^. Thus, organs sharing a close vascular and/or neurological anatomy, such as liver and spleen, may represent a mutual pathway to the protective effects related to RIPC.

In rat, liver and spleen are mainly connected through splenic vein, which is an important tributary of the extrahepatic part of portal vein. It has been reported that inflammatory mediators migrate to hepatic parenchyma during post-ischemic reperfusion^8^, indicating a potential hepatosplenic route for the transfer of humoral factors released after RIPC. On the other hand, the possible contribution of a splenic neural pathway during RIPC is more difficult to hypothesize. In a previous study by Lieder *et al*.^26^, splenic denervation abrogated cardioprotection by RIPC in rats, whereas pharmacological activation of muscarinic receptors in an isolated perfused spleen induced the release of cardioprotective factors, supporting a vago-splenic axis. Therefore, we suggest that this neurological signaling could also be activated by our sIPC procedure; however, further studies are needed to elucidate such a hepatoprotective effect.

In a clinical scenario, the consequences of hepatic IRI are generally traduced as tissue poorly functioning after major resections or transplantation^2^, but also as a systemic inflammatory response with multi-organ involvement^27^. In the present study, we showed that spleen is significantly affected by liver IRI. Similar with our findings, previous reports also demonstrated that hepatic IRI induced remote oxidative damage on spleen^28^ ^,^ ^29^.

In our experiment, splenic injury was probably a direct consequence of the release of ROS into circulation during hepatic reperfusion and the depletion of antioxidant enzymes was an indirect effect due to increased prooxidant mediators. We also assume that the model used for partial hepatic ischemia caused a certain degree of splenic congestion, which may have induced splenic production of cytokines, macrophage activation and additional injury to the spleen as suggested previously^8^.

In sIPC group, intermittent occlusion of splenic artery acted as a local IPC to spleen, reducing TBARS levels and attenuating the remote harmful effects from liver IRI. This protection may have been mediated in part through activation/inhibition of transcription factors with subsequent increase of endogenous antioxidant enzymes, such as CAT, GPx and GSH-Rd.

In the sIPC group, although liver and spleen had reduced levels of TBARS associated with increased activities of CAT, GPX and GSH, the favorable outcome appeared to be more effective on the splenic tissue. A possible explanation for this finding is that postischemic dysfunction of hepatic perfusion^2^ may have impaired the passage of protective humoral factors, reducing the benefits of sIPC in liver. Furthermore, we suggest that effectiveness of RIPC may depend on which organ is applied. In our study, the spleen was affected by hepatic IRI and it is feasible that protective mediators expressed in splenic parenchyma were consumed locally to confront the damage *in situ*, which diminished their action at distance.

## Conclusion

sIPC attenuated hepatic and splenic IRI-induced oxidative stress by enhancing the activity of endogenous antioxidant enzymes, such as CAT, GPx and GSH-Rd. Further studies are needed to confirm these results and elucidate the mechanisms by which sIPC establishes tissue protection.
